# Socio-cultural brain reprogramming–The uniqueness of human cognition

**DOI:** 10.3389/fnhum.2024.1331213

**Published:** 2024-03-12

**Authors:** Daniel Żuromski, Anita Pacholik-Żuromska

**Affiliations:** Department of Cognitive Science, Institute of Information and Communication Research, Nicolaus Copernicus University, Toruń, Poland

**Keywords:** language, evolution, culture, tool, embodied cognition, human uniqueness, social skill acquisition

## Introduction

*It takes more than one human brain to create a human mind*.

Barrett (2017)

*We are smart, but not because we stand on the shoulders of giants or are giants ourselves. We stand on the shoulders of a very large pyramid of hobbits*.

Henrich (2015)

It is difficult to say something unique about the human uniqueness, as it is currently one of the key research problems of contemporary science and the empirical findings are coming from many different disciplines considering different levels of human constitution: from neural information processing to social interactions. In the frame of neurosciences one of the approaches addressing this question involves a comparative analysis of brain volumes among various primates (Dunbar, 1998). For instance, chimpanzees exhibit a brain size roughly one-third that of humans. However, relying solely on brain volume as a metric may not offer the most accurate insight into what sets us apart from other hominins. Recent advancements in our understanding of other Homo species, such as Homo neanderthalensis and Homo heidelbergensis, reveal less pronounced differences in brain volume. Furthermore, the emergence of Homo sapiens dates back ~200,000 to 300,000 years, but a pivotal moment in our cognitive evolution becomes apparent in the fossil record only around 80,000 years ago. This significant cognitive leap suggests that factors beyond sheer brain size play a crucial role in differentiating human cognition from that of our hominin relatives (Hare, 2017). Continuing the research about human uniqueness, at the beginning of 21th century neuroscientists have identified astrocytes that are exclusive to the human brain (Robertson, 2014). These discoveries have led to fresh insights into the intricate local and global structures of the human brain, unveiling their profound associations with the advanced cognitive abilities unique to humans (Oberheim et al., 2006; Zhang and Barres, 2013).

We believe that the question of what makes us humans unique makes sense only in the light of evolution, as Dobzhansky (1973) notably stated: “Nothing in biology makes sense except in the light of evolution”. In other words, human uniqueness is a result of evolution and an aggregate of skills developed in the phylogenesis. We claim that the use of tools and the development of language—which can be also considered as a kind of a tool—had a crucial impact on where humans are now on the ladder of evolution. Human beings are able to enhance their natural skills by an incorporation of artifacts and the use of substitutes to create an augmented reality (Clark, 2008). Cognitive processes can be augmented or extended through the use of tools, technology, and other external aids, which should extend our minds beyond its natural boundaries adding new capacities like a larger memory by the use of external sources (e.g., a calendar in the cell phone). This super-ability challenges the traditional view of the mind as being strictly localized within the skull. “Human minds and bodies are essentially open to episodes of deep and transformative restructuring in which new equipment (both physical and “mental”) can become quite literally incorporated into the thinking and acting systems that we identify as our minds and bodies” (Clark, 2008, p. 30–31). We believe that this openness to transformation distinguishes us from other anthropoids. In other words, the use of artifacts as tools transforming the body and the language as a tool transforming the mind has an significant role in human evolution.

## Artifacts as tools transforming the body

Theories of the embodied mind hold that the body plays an important role in cognition. According to the thesis about embodied cognition, minds need a body to test and explore the possibilities for incorporating new resources and structures into their embodied acting (Clark, 2008, p. 42). Embodied cognition is intricately tied to adaptive processes, rendering it susceptible to the forces of evolution. Anderson (2007) posits that this evolutionary perspective on cognition is instrumental in unraveling its purpose. Consequently, the underlying implication is that cognition primarily serves the imperative of survival. Notably, cognition is an attribute of organisms that have undergone development within specific environmental parameters. Hence, cognition has progressed hand in hand with the evolution of sensory organs and the nervous system, both crucial for enabling cognitive processes and subsequent actions in a particular ecological setting. In essence, cognition is finely tuned to fit the ecological niche of each distinct species. To encapsulate Anderson's perspective succinctly, “cognition represents a collection of tools with a specialized and interdependent function that has evolved due to their adaptability in nature” (Anderson, 2007, p. 67).

What makes human unique in the use of tools is their unparalleled adaptability and willingness to integrate a diverse array of intricate tools and artifacts into their embodied, perceptual, cognitive, and affective systems (Heersmink, 2022). During tool manipulation, a dynamic relationship emerges among the body, task, and environment, resulting in the transformation of the body-only system into a body-plus-tool system (Mangalam and Fragaszy, 2016). The incorporation of tools into the body schema significantly extends both the physical and sensory reach of human capabilities. Different types of artifacts influence the body schema and expand the horizons of perception and action (Black, 2014). The increasing development of rehabilitative robotic technologies highlights that tools serve not only the purpose of convenience but are also employed in rehabilitation. For instance, exoskeletons aid in addressing the prevalence of mobility impairments stemming from spinal cord injuries and cerebrovascular accidents (Butnaru, 2021).

What is interesting is that the use of tools and the ability to communicate are not exclusive to humans. Crows, for instance, have adapted to urban environments, acquiring knowledge about humans and passing it down to subsequent generations, thereby developing a form of culture (Cornell et al., 2012). Notably, among the Corvus species, the New Caledonian crow has demonstrated the ability to use tools (Bayern et al., 2018). However, other avian species, as well as apes, sea otters, elephants, dolphins, among others, also exhibit tool use (Beck, 1980; Bentley-Condit and Smith, 2010).

Despite the widespread occurrence of tool use in various species, the skills we share with other organisms become unique to humans due to further evolutionary development. Advancements in representational abilities, executive functioning, sensorimotor capabilities, and cognitive skills related to collaborative culture may play a pivotal role in influencing the evolution of tool use (Seed and Byrne, 2010). Modern humans' ability to use tools is closely linked to the evolution of cognitive skills. For example the increased working memory influences various cognitive domains such as theory of mind, symbolic thought, analogical reasoning, and planning (Seed and Byrne, 2010). Our behavior, mind and cognitive abilities are the result of evolution in the broad sense of the word, including both genetic and cultural evolution. A large part of contemporary research and discussions on human uniqueness focuses on the evolution in the cultural aspect (Colagè and d'Errico, 2018; Bender, 2020). The social contexts, shaped by The Social Intelligence Hypothesis (critical view Barrett, 2016), plays a pivotal role in the development of cognitive abilities (e.g., Machiavellian intelligence—Byrne and Whiten, 1988; The social brain hypothesis—Dunbar, 1998; “primate politics”—De Waal, 1982). Of course, non-human primates are inherently social animals, relying on advanced social cognitive abilities, including a rudimentary form of theory of mind, to navigate their complex social environments. Despite the shared social nature of Homo sapiens and nonhuman apes, humans stand out as “The ultra-social animal”, as aptly described by Tomasello (2014). Human cognitive abilities, specifically those related to learning, cooperation, and communication are primarily geared toward the development of culture and processes associated with cultural evolution (Herrmann et al., 2007, 2010; Mesoudi, 2011; Lewens, 2015) as well as cumulative cultural evolution (Tomasello, 1999; Mesoudi, 2016; Mesoudi and Thornton, 2018). This has facilitated the accumulation, transmission, and modification of knowledge, skills, and technological innovations not just in face-to-face interactions but also across generations. Consequently, human creations are no longer solely the product of individual brains but rather of a collective intelligence (Henrich, 2015; Muthukrishna and Henrich, 2016; Henrich and Muthukrishna, 2023; Henrich et al., 2023).

## Language as a tool transforming the mind

“Cultural evolution has shown us that one word can be worth a thousand genes” (Szathmáry and Számadó, 2008). In this sense, language serves not only as a means of communication and expression of mental states, but also as a complex cultural artifact that has evolved over time through cumulative cultural processes (Tomasello, 2009; cf. Sterelny, 2014). Tomasello and Rakoczy (2003) outlines a specific hypothesis regarding the transformative role of language in human cognitive development. He suggests that language serves two primary functions: facilitating communication and enabling cognitive representation. He underscores the close interrelation between these functions, emphasizing the significance of uniquely human social cognition in the process. According to Tomasello and Rakoczy (2003), the key lies in understanding how this distinct social cognition enables children to learn and utilize linguistic symbols for interpersonal communication, a process that typically begins around the second year of life. Over time, these interpersonal communication tools are internalized and start being used intrapersonally, ultimately becoming the primary medium for specific types of human cognition. Acquiring language requires specific cognitive abilities, such as joint attention and the comprehension of intentions, as well as active engagement in social experiences and participation in specific social practices, such as scenes of joint attention (Kern and Moll, 2017).

Of course, non-human primates are also social animals. At the core of their strategies for coping in a complex social environment lie advanced cognitive abilities related to social cognition, such as, for example, theory of mind to some extent. One hypothesis suggests that great apes possess cognitive abilities similar to humans, but their primary function is competition rather than cooperation (cf. Cheney and Seyfarth, 2007; Tomasello, 2023). In other words, their ability to read the minds of others may be used in the context of cooperation, but the main goal is competition. As support for these theories, it is pointed out that great apes more effectively handle certain tasks when they are related to competition than when they involve cooperation or communication (Hare et al., 2001; Hare and Tomasello, 2004).

Language as a tool fulfills an operative psychological function, as its internalization within the context of social interactions facilitates the semiotic mediation of mental processes (cf. Trevarthen, 1998; Brinck and Liljeenfors, 2013). Here, a linguistic symbol is regarded as a means that transforms elementary mental functions into higher functions (Wertsch, 1985; Tomasello, 1999). Clark (1998, 2008) highlights the importance of understanding the role of language in shaping human cognition and behavior within the context of our dynamic and adaptive interaction with the environment. This dynamism includes both its transformative character and the socio-cultural aspects. “While numerous species engage in representation and communication, humans uniquely employ a single system for both functions. Human language serves dual roles: it functions as an intra-individual representational system and, simultaneously, as an inter-individual communication system” (Astington and Baird, 2005, p. 6). Communicative function of language and its cognitive function are two aspects of one process. For instance, the development of abilities such as joint attention enables social interactions that influence the capacity to adopt perspectives and understand communicative intentions in specific language discourses.

The genesis of higher psychological functions is rooted in language-based social interactions. A sign integrated into the natural developmental process, embraced by the child within the cultural developmental trajectory, restructures the cognitive system and its basic mental functions—such as attention, memory, perception, and thinking—into higher functions such as metacognition (cf. Żuromski et al., 2022). In the process of internalization, language undergoes a fundamental shift in function, initially serving as a means for controlling others' behavior in the social speech and later transforming into an executive and self-regulation function for cognitive processes and behavior in the private and inner speech (Wertsch, 1985; Vygotsky, 1987). In line with contemporary perspectives, Fernyhough (2008, 2009) posits that both the sequence of explanations and Vygotsky's exploration of verbal thinking provide insights into the processes of dialogical thinking, rooted in external (social) dialogue (Vygotsky, 1997, 1999). In dialogical exchange of beliefs, the connection between self and others is contained. The inherent tension between “me” and “other” serves as a natural force shaping the development of an autonomous and self-aware subject. An outcome of interaction through language is metacognition, which evolves alongside language acquisition and initial social interactions (Heyes, 2016; Heyes et al., 2020).

To conclude, on an evolutionary time scale, our cognitive abilities and our minds are the result of evolution in a broad sense, both genetic and cultural. In this first sense, much of our cognitive endowment is in fact inherited from sharing a common ancestor with apes. The consequences of the processes of the natural and cultural evolution encompass not only innovations like tools, technology, institutions, social norms, advanced mathematical notations, and intricate scientific theories but also specific and distinctive cognitive abilities termed as “cognitive gadgets” (Heyes, 2018a). These cognitive gadgets include language (Tomasello, 2005; Christiansen and Chater, 2016; Sterelny, 2016; Planer and Sterelny, 2021), theory of mind (Heyes and Frith, 2014; Moore, 2020), empathy (Heyes, 2018b), and emotions (Barrett, 2017; Lindquist et al., 2022). These socio-cultural cognitive abilities are transformative—they alter abilities already shared with other nonhuman apes, such as causal understanding, spatial memory, and tool use (Moll, 2018). In this context, primatology, evolutionary psychology, comparative psychology, and neuroscience provide insights into the evolution of human cognitive abilities. This knowledge extends beyond our own species to encompass other members of the Homo genus (e.g., Homo erectus or Homo neanderthalensis) as well as great apes (chimpanzees, orangutans, etc.). As Herrmann et al. (2007, p. 1361) suggest: “Humans' especially powerful skills of social-cultural cognition early in ontogeny serve as a kind of ‘bootstrap' for the distinctively complex development of human cognition in general”. Referring to the processes of cumulative cultural evolution, which enable the accumulation and modification of innovations across generations, we can explain how humans are involved not only in creating tools but also in developing advanced technologies for tool creation. The various forms of human activity find their basis in distinctly human cognitive mechanisms—the fundamental processes of the human mind. Throughout the course of cultural evolution, these underlying cognitive mechanisms have undergone transformation (Heyes, 2019, p. 1).

## Author contributions

DŻ: Writing – review & editing, Writing – original draft. AP-Ż: Writing – review & editing, Writing – original draft.
